# Socioeconomic and demographic correlates of child nutritional status in Nepal: an investigation of heterogeneous effects using quantile regression

**DOI:** 10.1186/s12992-022-00834-4

**Published:** 2022-04-20

**Authors:** Umesh Prasad Bhusal, Vishnu Prasad Sapkota

**Affiliations:** 1 Public Health and Social Protection Professional, Kathmandu, Nepal; 2 Department of Economics, Nepal Commerce Campus, Tribhuvan University, New Baneshwor, Kathmandu, Nepal

**Keywords:** Quantile regression, Nutrition, Height-for-age z scores, MICS, Nepal, SDGs, UHC

## Abstract

**Background:**

Child undernutrition continues to be a major public health problem in many countries, including Nepal. The repercussions of undernutrition are not only limited to the affected children and families but also transcend to the national and global economy. Earlier studies from Nepal have predominantly used either ordinary least squares (OLS) regression or binary regression to analyse the socioeconomic and demographic correlates of the nutritional outcome. In this study, quantile regression was used to understand a complete and more precise estimate of the effects of the covariates on the child nutritional status.

**Methods:**

This study was based on the most recent nationally representative Nepal Multiple Indicator Cluster Survey (MICS) 2019. Height-for-age z scores (HAZ) were used as an indicator for assessing the nutritional status of under-five children. Quantile regression was used to examine the heterogeneous association of covariates with conditional HAZ distribution across the different quantiles (0.10, 0.30, 0.50, 0.85). As a comparison, the effects of covariates at conditional mean of HAZ using OLS regression was also analysed. The graphs were plotted to visualize the changes in the coefficients for each regressor across the entire conditional HAZ distribution.

**Results:**

Age of children, sex of children, province and wealth had a consistent and statistically significant association with HAZ in both OLS and quantile regression. Improved toilet facility was positively correlated with HAZ at the lower tails (tenth and thirtieth percentiles). Ethnicity (Janajati and Newer) was positively correlated with HAZ at the lower tail (thirtieth percentile) and mean (OLS regression). Maternal education was a significant predictor of improved height-for-age across conditional quantiles, except at the tenth percentile. Maternal age, number of under-five children in household, number of household members, and improved source of drinking water showed heterogeneous effects across different quantiles of conditional HAZ distribution.

**Conclusion:**

Use of quantile regression approach showed that the effect of different factors differed across the conditional distribution of HAZ. Policymakers should consider the heterogeneous effect of different factors on HAZ so that the targeted intervention could be implemented to maximize the nutritional benefits to children.

**Supplementary Information:**

The online version contains supplementary material available at 10.1186/s12992-022-00834-4.

## Background

Proper nutrition in-utero, early childhood is vital for physical and mental growth and development [1]. Children need adequate nutrients (carbohydrates, proteins, fats) and micronutrients (minerals, vitamins) for proper body functioning, immune system development, and overall growth [2]. In comparison to well-nourished children, undernourished children usually have a compromised immune system, decreased intellectual and cognitive abilities, increased risk of mortality and morbidities, a decreased chance of attaining their optimum potential height and productivity [1–3]. The repercussions of undernutrition are not only limited to the directly affected children and families but also transcend to the national and global economy [4].

Globally, in 2020 estimated number of under-five children stunted (too short for age) and wasted (too thin for height) was 149.2 million and 45.4 million, respectively [1]. The figure corresponds to 22% of under-five children affected by stunting and 6.7% affected by wasting. The Asian countries bear an unfair share of this problem, as 53% of under-five children affected by stunting and 70% of under-five children affected by wasting were from Asia [1]. About half of deaths among under-five children are linked to undernutrition [4, 5]. Since these estimates were largely based on data collected before 2020, the current situation could be worse owing to the potential impact of the Covid-19 pandemic [1].

In Nepal, the nutritional status of under-five children has improved since 1996. In 1996, 57% of under-five children were too short for age compared to 36% in 2016 [6]. As per the recent household survey in 2019, the figure has further decreased to 31.5% [7]. However, the progress in reducing stunting is not up to par with the World Health Assembly target of reducing stunting by 3.9% per year or 40% by 2025 from the baseline of 2010 [8]. So, child undernutrition continues to be a major public health problem in Nepal.

Government of Nepal (GoN) is committed to improving the nutritional status of women, children and adolescents and achieving Sustainable Development Goals (SDGs) targets by 2030 [9]. Target 2.2 of SDGs aims to end all forms of malnutrition by 2030 [10]. Nutrition specific policies and strategies are guided mainly by National Health Policy 2019, Nepal Health Sector Strategy (2016–2022), National Nutrition Strategy 2020, and Multi-Sector Nutrition Plan I (2013–2017) and II (2018–2022). GoN is also committed to the ‘scaling up nutrition’ global movement that calls for multi-sectoral actions for improved nutrition during the first 1000 days of life [9].

Height-for-age reflects cumulative linear growth, which could be affected by chronic nutritional deprivation and chronic or frequent illness [11]. In contrast to height, the body weight of children is sensitive to short-term alteration in diet and illness [3]. So, height-for-age is a more commonly used indicator of child nutrition since it captures the effect of chronic undernutrition if any. Studies from a wide range of countries have shown that the nutritional status of under-five children is affected by a multitude of factors, ranging from child, maternal, socioeconomic, demographic to household hygiene and sanitation [12–18]. These studies have predominantly used either the ordinary least squares (OLS) regression or binary regression to analyse the determinants of nutritional outcome. Since OLS regression estimates the effect of various factors at the conditional mean of the outcome variable, the result may over-or underestimate the actual effect of the covariates along the different quantiles of the distribution of outcome variable, especially in the case with anthropometric data [3, 19–22]. Similarly, since binary regression models treat observations that are below or above a threshold level equally, the intensity of the deviations from that threshold level cannot be taken into account [3, 21, 23].

In contrast, quantile regression approach provides a complete and more precise estimate of the effects of the covariates on the outcome variable without dichotomization and loss of information [12, 21, 22, 24, 25]. This approach yields optimal estimates of regression coefficients even in the presence of outliers and non-normal distribution of outcome variable [19, 21, 22, 24, 26]. Researchers prefer outcome variable to be on a continuous measurement scale with an argument that dichotomizing variables unnecessarily weakens the power of statistical tests [27]. Earlier studies from Nepal that aimed to analyse the factors affecting nutritional status of children have mostly used linear regression (OLS), binary regression or linear probability models that were inadequate to analyse the full scenario of effects [16–18, 28–32]. However, the use of quantile regression in global nutrition literature is growing due to the methodological advantages over standard linear or binary regression as mentioned above.

The objective of this study was to analyse the socioeconomic and demographic correlates of nutritional status of under-five children in Nepal using data from the most recent nationally representative household survey. Height-for-age z scores (HAZ) were used as an indicator of nutrition status. This study adds to the current body of literature in Nepal by using a quantile regression approach to investigate heterogeneous effects of covariates at the various points of the conditional HAZ distribution. The study provides evidence to policymakers and planners to design the specific intervention along the quantiles of height-for-age distribution so that SDGs target of ending all forms of malnutrition could be achieved by 2030, together with achieving universal health coverage (UHC).

## Methods

### Data source and sampling design

This study was based on Nepal Multiple Indicator Cluster Survey (MICS) 2019, a nationally representative cross-sectional household survey. The survey aims to monitor the situation of women and children by collecting information on health, education, social protection, environment along with socioeconomic, demographic, and geographic characteristics. The survey was conducted by the Central Bureau of Statistics and UNICEF. The sampling frame of the 2019 survey round was based on National Population and Housing Census 2011. The sampling frame comprised a list of all census wards constructed in 2011. The list of census wards was updated in 2018 to account for the changes in the administrative structure of Nepal during federalization. To establish a representative sample of households at the national and sub-national level, the survey used a multistage, stratified, cluster probability sampling design. The main sampling strata were defined as urban and rural areas of each province. The sample of households was obtained in two stages: (i) in each stratum, a specified number of census enumeration areas (EAs) or clusters were selected systematically with probability proportional to size. After that, the listing of the household was carried out for the selected EAs (ii) the sample of households was selected from the sampled EAs using a systematic random sampling method. In total, 25 households per sampled EA were obtained. From a total of 512 EAs, 12,800 households were selected. Out of which, number of households, women (15–49 years) and men (15–49 years) successfully interviewed were 12,655, 14,805 and 5501, respectively. Further detail about the MICS design is available in the survey findings report [7].

### Study population

This study extracted information about HAZ of 6469 under-five children obtained by interviewing mothers or primary caretakers. After removing 181 observations with incomplete information about the variables selected for this study, the final sample was 6288 under-five children.

### Study variables

The outcome variable of interest was HAZ of under-five children (0–59 months) measured on a continuous scale. Height-for-age measures children’s height relative to their age. To calculate HAZ: the difference between child’s height and median value for the reference population for the corresponding age and sex is divided by the standard deviation (SD) of the reference population [11]. Nepal MICS 2019 used World Health Organization (WHO) growth standards as the reference population. So, HAZ is height-for-age expressed in SD units (z-scores) from the median of the WHO growth standards obtained from the reference population. A wide range of policy-relevant variables commonly used in literature as the predictors of nutritional status of children were taken as the independent variables [2, 3, 15, 21, 33], including UNICEF framework depicted in Fenske et al. [33]. The final list of variables included in this study, however, was constrained by the data available in the Nepal MICS 2019. The list of variables and their measurement is presented in Table 1. Few variables need additional description. Source of drinking water and type of toilet facility were categorized into improved and unimproved using WHO guidelines [34]. More than 100 castes listed during the survey were allocated into four categories using the guideline from Population Monograph of Nepal 2014 [35]. We used wealth index quintile available in Nepal MICS 2019 dataset as a measure of living standard. The popular methods to quantify household living standard are consumption, income and wealth [11]. However, due to absence of such measures in MICS, wealth index was used as a proxy variable for living standard. To construct the wealth index, MICS employed principal components analysis using data on the ownership of consumer goods, dwelling characteristics, water, sanitation, and other assets/durables that were related to the household’s wealth [7].

**Table 1:** List of variables and their measurement

Variable	Measurement
Height-for-age z-score (HAZ)	Continuous variable measured as per WHO standards
**Child characteristics**	
Child’s age	Age of children in months
Child’s sex	Categorized as: female; male
Birth order	Categorized as: first; second and third; fourth and above
**Mother’s characteristics**	
Mother’s age at birth	Categorized as: less than 20 years; 20–34 years; more than 34 years
Education status of mother	Categorized as: no formal education; primary education (grade1-5); secondary education (grade 6–10); higher secondary and above (grade 11 and above)
**Household characteristics**	
Number of under-five children in household	Categorized as: one; two; three; four or more
Number of household members	Categorized as: two to five members; six to eight members; nine or more members
Source of drinking water	Categorized as: improved; unimproved
Type of toilet facility	Categorized as: improved; unimproved
Ethnicity	Categorized as: Brahmin, Chhetri and Madhesi; Janajati and Newar; Dalit and Muslim; Others (eg. Marwadi, Bangali)
Residence	Categorized as: rural; urban
Province	Categorized as: Province 1; Madhesh province; Bagmati province; Gandaki province; Lumbini province; Karnali province; Sudurpaschim province
Wealth index quintile	Categorized as: poorest; poor; middle; richer; richest

### Method of analysis

The socioeconomic and demographic characteristics of the study population (under-five children) were described by mean (SD) for continuous variable and frequency (percentage) for categorical variable.

Quantile regression modelling was employed to investigate the correlates of conditional quantiles of HAZ. Since quantile regression models the conditional quantiles of a dependent variable as a linear function of the independent variables, this approach allowed us to examine the heterogeneous association of covariates across the different quantiles of conditional HAZ distribution (0.10, 0.30, 0.50, 0.85). As per the cumulative probability distribution of HAZ, the selected quantiles correspond approximately to the proportion of the children in our sample with HAZ < -3 SD (12%), < -2 SD (32%), median (50%), and < 0 SD (85%), respectively.

We estimated quantile regression of the following form, as shown in Eq. 1

1 $${y}_{i}={x}_{i}^{^{\prime}}{\beta }_{\tau }+{\varepsilon }_{i}$$

where, ${\beta }_{\tau }$ is the vector of unknown parameters associated with the ${\tau }^{th}$ quantile. $x$ is the vector of socioeconomic and demographic characteristics.

The ${\tau }^{th}$ quantile regression estimator of ${\beta }_{\tau }$ was estimated by minimizing the following objective function, as shown in Eq. 2

2 $$\mathrm{\rm T}\left({\beta }_{\tau }\right)={\Sigma }_{i:{y}_{i}\ge {x}_{i}^{^{\prime}} \beta }^{N}\tau \left|{y}_{i}-{x}_{i}^{^{\prime}} \beta {}_{\tau }\right|+ {\Sigma }_{i:{y}_{i}<{x}_{i}^{^{\prime}} \beta }^{N}(1-\tau )\left|{y}_{i}-{x}_{i}^{^{\prime}} {\beta }_{\tau }\right|$$

Here, 0< $$\tau$$  < 1.

Analyses were performed in Stata 16.0 (StataCorp).

**Fig. 1:**
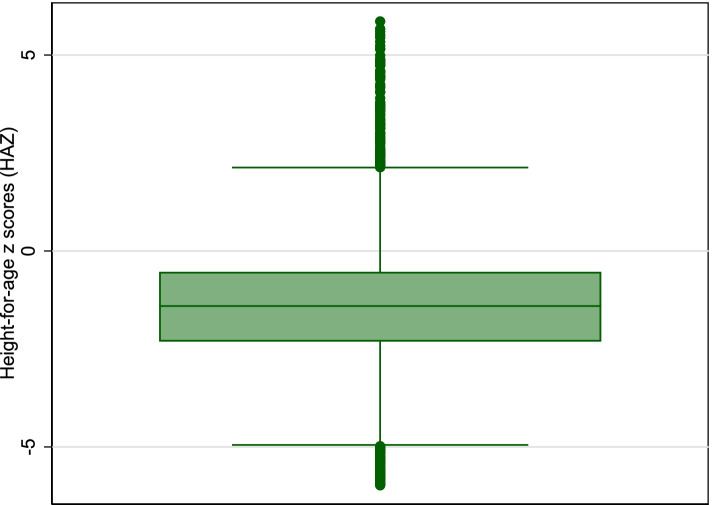
Boxplot of Height-for-age z scores (HAZ)

Quantile regression is a non-parametric method, so it does not require specific distributional assumptions on residuals. This is an advantage over the OLS regression at conditional mean, where conditional mean itself is affected by the extreme values of the residuals. However, the quantile regression estimates are robust to such extreme values or specific distribution of residuals. Multicollinearity among covariates was examined using variance inflation factors (VIFs) before building multivariable regression models. Covariates with VIFs < 3.0 were included in the multivariable regression. The ‘preceding birth interval’ was removed from the analysis due to high VIF value. The details of the VIFs of variables included in the models are provided in Supplementary Table 1. The non-normal distribution of HAZ (positively skewed) evident from the boxplot in Fig. 1 provided a rationale for using quantile regression.

Test for heteroskedasticity (constant conditional variance assumption of OLS regression) was done using Breusch-Pagan test statistic. We find that the test statistic is significantly different than zero (*p*-value < 0.001), which also justified the use of quantile regression. We have reported the finding from the OLS regression as well to compare it with the finding from quantile regression. The reported confidence intervals (CI) in OLS regression correspond to heteroskedasticity-consistent standard error of estimators [36]. The graphs were plotted to visualize the changes in the coefficients for each regressor across the entire conditional HAZ distribution. All analyses were weighted using the sampling weights available in the Nepal MICS 2019 dataset.

## Results

### Descriptive summary

Table 2 presents socioeconomic and demographic characteristics of the study sample of under-five children. The mean HAZ was -1.3, meaning Nepalese children were, on average, 1.3 SD shorter than the WHO reference population. The mean age of the children was 30.4 months. More than half (52.7%) of the children were male. Most of the children were either first born (42.4%), or second and third born (46.4%). Majority of the households had one child aged below five (60.9%), followed by two (30.8%), three (6.6%), and four or more (1.7%). Majority of mothers were aged 20–34 years old (76.3%), had received secondary education (38.6%). About half of the household had two to five family members (51.4%), followed by six to eight (35.2%). More than 90% of the households had improved source of drinking water and toilet facility. In terms of ethnicity, majority of the children belonged to upper or middle caste with 15.5% belonged to lower caste (Dalit and Muslim). Nearly 65% of the children were from urban residence. Regarding province, most children belonged to Madhesh (23.1%), followed by Bagmati (18.9%), Lumbini (18.6%), Province 1 (15.8%), Sudurpaschim (9.8%), Gandaki (7.3%) and Karnali (6.5%).

**Table 2:** Socioeconomic and demographic characteristics of study sample of under-five children, Nepal MICS 2019 (*N* = 6288)

Variables	Mean (SD) or frequency (%)
**Height-for-age z-scores (HAZ)**	-1.3 (1.5)
**Child characteristics**	
**Child’s age (months)**	30.4 (17.3)
**Child’s sex**	
Female	2976 (47.3)
Male	3312 (52.7)
**Birth order**	
First	2668 (42.4)
Second and third	2904 (46.2)
Fourth and above	716 (11.4)
**Mother’s characteristics**	
**Mother’s age at birth**	
< 20 years	1136 (18.1)
20–34 years	4800 (76.3)
> 34 years	352 (5.6)
**Education status of mother**	
No formal education	1563 (24.9)
Primary education (grade1-5)	964 (15.3)
Secondary education (grade 6–10)	2428 (38.6)
Higher secondary and above (grade 11 and above)	1333 (21.2)
**Household characteristics**	
**Number of under-five children in household**	
One	3831 (60.9)
Two	1936 (30.8)
Three	414 (6.6)
Four or more	107 (1.7)
**Number of household members**	
Two to five members	3230 (51.4)
Six to eight members	2215 (35.2)
Nine or more members	843 (13.4)
**Source of drinking water**	
Improved	5734 (91.2)
Unimproved	554 (8.8)
**Type of toilet facility**	
Improved	5819 (92.5)
Unimproved	469 (7.5)
**Ethnicity**	
Brahmin, Chhetri and Madhesi	2690 (42.8)
Janajati and Newar	2102 (33.4)
Dalit and Muslim	977 (15.5)
Others (eg. Marwadi, Bangali)	519 (8.3)
**Residence**	
Rural	2214 (35.2)
Urban	4074 (64.8)
**Province**	
Province 1	991 (15.8)
Madhesh province	1451 (23.1)
Bagmati province	1190 (18.9)
Gandaki province	459 (7.3)
Lumbini province	1169 (18.6)
Karnali province	412 (6.5)
Sudurpaschim province	616 (9.8)
**Wealth index quintile**	
Poorest	1458 (23.2)
Poor	1294 (20.6)
Middle	1274 (20.3)
Richer	1215 (19.3)
Richest	1047 (16.6)
**Total**	**6288 (100)**

*Abbreviation*: *SD* Standard Deviation, *MICS* Multiple Indicator Cluster Survey

### OLS and quantile regression

Table 3 presents coefficients from OLS regression and quantile regression at different points of conditional HAZ distribution (0.10, 0.30, 0.50, 0.85). The variables showed differential effects depending on whether the modelling was done at the conditional mean (OLS) or at the conditional quantiles of HAZ distribution. For variables that showed statistically significant association at conditional mean and all the conditional quantiles of HAZ, the strength of association was heterogeneous.

**Table 3:** Results of multivariable OLS regression and quantile regression for height-for-age z scores (HAZ) of under-five children, Nepal MICS 2019 (*N* = 6288)

Variables	Coefficients (95% CI)				
	**OLS**	**0.10 quantile**	**0.30 quantile**	**0.50 quantile**	**0.85 quantile**
**Child characteristics**					
**Child’s age (months)**	-0.02 (-0.023 to -0.018)***	-0.01 (-0.02 to -0.01)***	-0.01 (-0.02 to -0.01)***	-0.02 (-0.02 to -0.01)***	-0.03 (-0.03 to -0.02)***
**Child’s sex**					
Female					
Male	-0.17 (-0.25 to -0.08)***	-0.21 (-0.36 to -0.06)**	-0.15 (-0.24 to -0.06)**	-0.13 (-0.22 to -0.04)**	-0.12 (-0.22 to -0.01)*
**Birth order**					
First					
Second and third	-0.06 (-0.16 to 0.05)	-0.02 (-0.19 to 0.15)	-0.07 (-0.18 to 0.03)	-0.04 (-0.15 to 0.06)	-0.02 (-0.13 to 0.09)
Fourth and above	0.04 (-0.15 to 0.24)	0.10 (-0.20 to 0.39)	-0.05 (-0.25 to 0.15)	0.01 (-0.16 to 0.18)	0.13 (-0.07 to 0.33)
**Mother’s characteristics**					
**Mother’s age at birth**					
< 20 years					
20–34 years	0.10 (-0.03 to 0.24)	0.13 (-0.10 to 0.37)	0.14 (0.03 to 0.25)*	0.19 (0.04 to 0.35)*	-0.003 (-0.14 to 0.14)
More than 34 years	0.01 (-0.27 to 0.30))	-0.15 (-0.43 to 0.14)	0.02 (-0.25 to 0.29)	0.13 (-0.13 to 0.39)	-0.03 (-0.39 to 0.33)
**Education status of mother**					
No formal education					
Primary education (grade1-5)	-0.06 (-0.22 to 0.10)	-0.19 (-0.46 to 0.08)	-0.09 (-0.24 to 0.05)	0.02 (-0.13 to 0.17)	0.03 (-0.14 to 0.20)
Secondary education (grade 6–10)	0.04 (-0.10 to 0.19)	-0.001 (-0.25 to 0.25)	0.14 (0.01 to 0.26)*	0.12 (-0.0005 to 0.23)	-0.06 (-0.20 to 0.08)
Higher secondary and above (grade 11 and above)	0.25 (0.08 to 0.43)**	0.08 (-0.18 to 0.34)	0.34 (0.17 to 0.51)***	0.36 (0.21 to 0.52)***	0.20 (0.01 to 0.40)*
**Household characteristics**					
**Number of under-five children in household**					
One					
Two	-0.03 (-0.13 to 0.08)	-0.12 (-0.28 to 0.03)	-0.09 (-0.19 to 0.02)	0.01 (-0.09 to 0.11)	-0.03 (-0.15 to 0.09)
Three	0.03 (-0.22 to 0.28)	0.05 (-0.37 to 0.48)	-0.30 (-0.50 to -0.10)**	-0.16 (-0.43 to 0.10)	0.37 (0.11 to 0.64)**
Four or more	-0.23 (-0.57 to 0.10)	-0.33 (-1.19 to 0.52)	-0.13 (-0.59 to 0.33)	-0.21 (-0.54 to 0.11)	0.05 (-0.65 to 0.76)
**Number of household members**					
Two to five members					
Six to eight members	0.07 (-0.03 to 0.18)	0.08 (-0.07 to 0.24)	0.09 (-0.01 to 0.19)	0.06 (-0.04 to 0.17)	0.05 (-0.07 to 0.17)
Nine or more members	-0.13 (-0.33 to 0.06)	-0.24 (-0.50 to 0.02)	-0.09 (-0.26 to 0.07)	-0.17 (-0.33 to-0.01)*	-0.16 (-0.33 to 0.01)
**Source of drinking water**					
Improved					
Unimproved	0.02 (-0.16 to 0.20)	0.07 (-0.46 to 0.59)	0.003 (-0.21 to 0.21)	0.08 (-0.16 to 0.31)	0.19 (0.01 to 0.36)*
**Type of toilet facility**					
Improved					
Unimproved	-0.18 (-0.39 to 0.03)	-0.74 (-1.15 to -0.33)***	-0.37 (-0.64 to -0.11)**	-0.08 (-0.29 to 0.12)	0.09 (-0.32 to 0.51)
**Ethnicity**					
Brahmin, Chhetri and Madhesi					
Janajati and Newar	0.12 (0.02 to 0.23)*	0.08 (-0.10 to 0.26)	0.15 (0.04 to 0.27)**	0.10 (-0.004 to 0.21)	0.10 (-0.02 to 0.21)
Dalit and Muslim	0.05 (-0.08 to 0.18)	0.02 (-0.17 to 0.20)	0.03 (-0.10 to 0.17)	0.04 (-0.09 to 0.17)	0.10 (-0.04 to 0.24)
Others (eg. Marwadi, Bangali)	-0.18 (-0.40 to 0.05)	-0.73 (-1.02 to -0.43)***	-0.18 (-0.35 to -0.02)*	-0.17 (-0.39 to 0.06)	-0.07 (-0.39 to 0.24)
**Residence**					
Rural					
Urban	0.06 (-0.04 to 0.15)	0.06 (-0.10 to 0.22)	0.02 (-0.07 to 0.11)	0.08 (-0.01 to 0.17)	0.05 (-0.06 to 0.15)
**Province**					
Province 1					
Madhesh province	-0.24 (-0.41 to -0.06)**	-0.54 (-0.83 to -0.25)***	-0.21 (-0.37 to -0.05)*	-0.25 (-0.42 to -0.07)**	-0.08 (-0.29 to 0.13)
Bagmati province	-0.31 (-0.46 to -0.16)***	-0.27 (-0.51 to -0.04)*	-0.22 (-0.37 to -0.07)**	-0.24 (-0.41 to -0.08)**	-0.40 (-0.53 to -0.26)***
Gandaki province	-0.23 (-0.39 to -0.08)**	-0.10 (-0.34 to 0.14)	-0.12 (-0.26 to 0.01)	-0.17 (-0.31 to -0.03)*	-0.34 (-0.52 to -0.16)***
Lumbini province	-0.44 (-0.58 to -0.30)***	-0.29 (-0.49 to -0.08)**	-0.37 (-0.50 to -0.23)***	-0.39 (-0.53 to -0.25)***	-0.52 (-0.70 to -0.35)***
Karnali province	-0.54 (-0.71 to -0.36)***	-0.65 (-0.94 to -0.37)***	-0.52 (-0.71 to -0.34)***	-0.54 (-0.74 to -0.34)***	-0.58 (-0.79 to -0.38)***
Sudurpaschim province	-0.43 (-0.59 to -0.27)***	-0.31 (-0.55 to -0.07)*	-0.43 (-0.58 to -0.28)***	-0.50 (-0.66 to -0.34)***	-0.45 (-0.63 to -0.26)***
**Wealth index quintile**					
Poorest					
Poor	0.26 (0.11 to 0.40)***	0.46 (0.20 to 0.72)**	0.30 (0.16 to 0.44)***	0.23 (0.10 to 0.38)**	0.25 (0.11 to 0.39)***
Middle	0.32 (0.17 to 0.48)***	0.49 (0.24 to 0.75)***	0.26 (0.11 to 0.40)***	0.26 (0.11 to 0.40)**	0.28 (0.12 to 0.43)**
Richer	0.44 (0.28 to 0.60)***	0.66 (0.38 to 0.94)***	0.42 (0.27 to 0.57)***	0.40 (0.25 to 0.56)***	0.43 (0.27 to 0.59)***
Richest	0.59 (0.39 to 0.78)***	0.97 (0.68 to 1.26)***	0.65 (0.44 to 0.86)***	0.47 (0.27 to 0.68)***	0.59 (0.36 to 0.81)***

***p < 0.001; **p < 0.01; *p < 0.05

*Abbreviation*: *CI* Confidence Interval, *MICS* Multiple Indicator Cluster Survey, *OLS* Ordinary Least Square

OLS regression showed that age and sex of children, education status of mother, ethnicity, province and wealth had a statistically significant association at the conditional mean of HAZ. With the increase in one month in age of children, the HAZ decreased by an estimated 0.02 SD (95% CI: -0.023 to -0.018). Male children had an estimated 0.17 SD (95% CI: -0.25 to -0.08) disadvantage over female children in terms of HAZ. Children born to educated mothers (higher secondary and above) were on average 0.25 SD (95% CI: 0.08 to 0.43) taller compared with children born to mothers with no formal education. Similarly, children belonging to Janajati and Newar ethnicity were 0.12 SD (95% CI: 0.02 to 0.23) taller than children belonging to Brahmin, Chhetri and Madhesi. Regarding province, children from Karnali had the least HAZ, followed by Lumbini, Sudurpaschim, Bagmati, Madhesh and Gandaki (reference = Province 1). Regarding household wealth, children belonging to the richest wealth quintile were tallest, followed by richer, middle and poor (reference = poorest wealth quintile).

Quantile regression at 0.10 showed that age and sex of children, toilet facility, ethnicity, province and wealth had a statistically significant association with HAZ of children. Similarly, quantile regression at 0.30 showed that age and sex of the children, number of under-five children in a household, maternal age at birth, education status of mother, toilet facility, ethnicity, province and wealth had a statistically significant association with HAZ of children. Moreover, quantile regression at 0.50 (median regression) showed that age and sex of children, maternal age at birth, education status of mother, number of household members, province and wealth had a statistically significant association with HAZ of children. In addition, quantile regression at 0.85 showed that age and sex of children, number of under-five children in a household, education status of mother, availability of improved drinking facility, province and wealth had a statistically significant association with HAZ of children.

Improved toilet facility was statistically significant at the lower tail of HAZ distribution (0.10 and 0.30) only. As per the quantile regression at 0.10 and 0.30, children belonging to household without improved toilet facility were 0.73 SD (95% CI: -1.15 to -0.33) and 0.37 SD (95% CI: -0.64 to -0.11) shorter than those belonging to household with improved toilet facility, respectively. However, the effect of household sanitation on upper quantiles and mean (OLS) of HAZ was not statistically significant. The ethnicity of children was statistically significant in OLS regression and quantile regression at lower tails (0.10 and 0.30) of HAZ distribution. In comparison to Brahmin, Chhetri and Madhesi, children belonging to Janajati and Newar were taller by 0.12 SD in OLS and 0.15 SD in quantile regression at 0.30. In contrast, children belonging to minority ethnic group (Bangali, Marwadi) were shorter by 0.73 SD in quantile regression at 0.10 and 0.18 SD in quantile regression at 0.30. These finding shows mean-based model (OLS) was inadequate to capture the potential heterogeneous effects of risk factors along different points of conditional HAZ distribution.

Secondary and higher secondary education of mother had a significant and heterogeneous positive effect on HAZ of children in OLS and quantile regression, except at 0.10. The largest effect of higher secondary education was observed at 0.50 (0.36 SD), and the least effect at 0.10 (0.08 SD), which were markedly underestimated and overestimated, respectively, by OLS point estimate (0.25 SD).

Maternal age at birth (20–34 years) was significantly positively associated with HAZ at 0.30 and 0.50. Number of under-five children in household had a significant inverse relationship with HAZ at 0.30. Similarly, number of family members in a household had a significant negative effect on HAZ at 0.50. Availability of an improved source of drinking water was significantly positively associated with HAZ at 0.85.

Age, sex, province and wealth demonstrated statistically significant heterogeneous effects along the conditional quantiles and mean of HAZ. Regarding province, children belonging to Karnali were the shortest, followed by Sudurpaschim, Madhesh and Lumbini (reference= Province 1). The effect in Karnali was largest at 0.10 (-0.65 SD), and least at 0.30 (-0.52 SD), which was slightly underestimated and overestimated, respectively, by OLS point estimate (-0.54 SD). Children from Madhesh were the most disadvantaged group after Karnali at 0.10 quantile (-0.54 SD). Whereas, children from Sudurpaschim were the most disadvantaged group after Karnali at 0.30 quantile (-0.43 SD) and 0.50 quantile (-0.50 SD).

Household wealth status differentially affected the HAZ of children. There was a significant and heterogeneous positive association between wealth index quintile and HAZ, evident from the OLS and quantile regression estimates across all the conditional quantiles of HAZ. The strength of association was strongest at 0.10 and weakest at 0.50. At any given quantile, HAZ of children increased almost monotonically in moving from poorest to the richest wealth quintile.

**Fig. 2:**
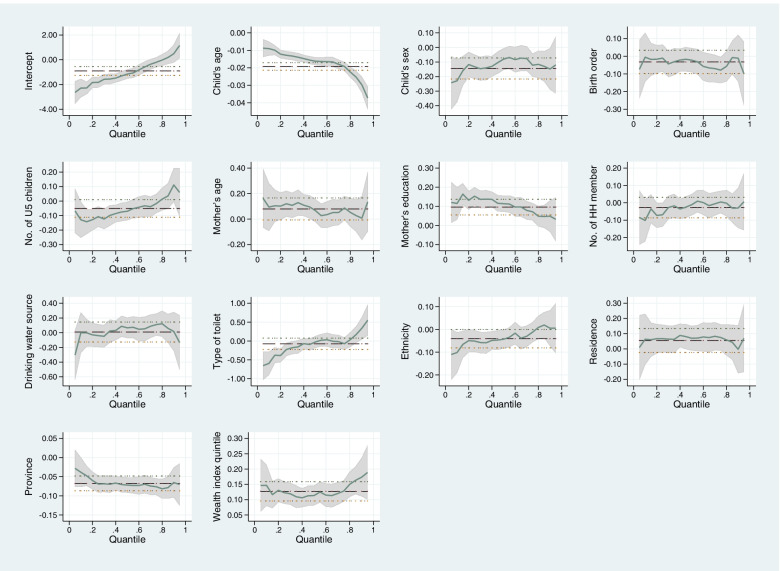
Plot of estimated coefficients from OLS regression and quantile regression. Here, *x-axis* denotes conditional quantiles of HAZ and *y-axis* denotes estimated coefficients

Figure 2 provides plot of estimated coefficients across conditional quantiles of HAZ. It illustrates the heterogeneous effect of the socioeconomic and demographic variables along the entire conditional HAZ distribution. In Fig. 2, the mean effect and corresponding CI obtained by OLS regression are denoted by dotted lines. Whereas, the effects and corresponding CIs obtained by quantile regression are denoted by green line and grey shaded area. Here, the coefficient (CI) obtained from OLS is constant across the quantiles. In contrast, the coefficient (CI) obtained from quantile regression varies with the location in the HAZ distribution.

## Discussion

This study analysed the socioeconomic and demographic correlates of nutritional status of under-five children in Nepal using quantile regression. This approach allowed us to examine heterogeneous effects of covariates along the conditional quantiles of HAZ distribution. As a comparison, we also analysed the effects of covariates at the conditional mean of HAZ using OLS regression. Coefficients obtained from OLS and quantile regression were plotted for each covariate to visualize the varying effects of covariates along different points of conditional HAZ distribution. Data from the most recent nationally representative household survey (Nepal MICS 2019) was used.

We found that age of children, sex of children, province and wealth had a consistent and statistically significant association with HAZ at every conditional quantile and mean. Availability of improved toilet facility was positively correlated with HAZ at the lower tails (tenth and thirtieth percentiles). This association was not evident in the OLS regression. Belonging to Janajati and Newer was positively correlated with HAZ at the lower tail (thirtieth percentile) and mean (OLS regression). Higher secondary education of mother was a significant predictor of improved height-for-age at every conditional quantile and mean, except at tenth percentile. Maternal age (20 to 34 years) was significantly correlated with improved HAZ at thirtieth and fiftieth percentiles. Children of a family with three under-five children were predicted to have a lower HAZ in comparison to a family with only one under-five child, at thirtieth percentile. Similarly, children of a family with nine or more family members were predicted to have a lower HAZ in comparison to a family with five or fewer family members, at fiftieth percentile. In addition, availability of an improved source of drinking water in household was significantly associated with improved HAZ, at eighty-fifth percentile.

Age of children was found to have a negative association with HAZ at mean and selected quantiles. This result is consistent with findings from similar studies conducted in Egypt, Bangladesh, Ghana, India, Pakistan, and Eswatini [3, 12–14, 19, 21, 33]. The association could be linked to inadequate complementary feeding practices and exposure to infections as the children get older in resource-poor settings [13, 37, 38]. In comparison to female children, male children were shorter. This finding corroborates the findings obtained from studies conducted in other countries [3, 14, 19, 24, 33]. A systematic analysis of sex differences in height-for-age among children less than five years of age from 10 sub-Saharan Africa has hypothesized one of the reasons as biological [39], as boys were generally found to be more susceptible to infectious diseases [19, 40]. However, the specific reason behind the sex differential in height of children is yet to be properly understood [40].

In line with the findings from other studies [3, 12–14, 19, 21, 24, 33, 38], we found that maternal education (secondary/higher secondary) was positively associated with HAZ of children. In studies from various settings, educated mothers were found to have a better knowledge about nutrition, childcare, child feeding, hygiene, risk associated with poor diet, and healthcare practices in comparison to non-educated counterparts, which could have positively influenced growth and development of children [3, 13, 14, 18, 19, 41, 42]. Similar to Fenske et al. [33], Mahmood et al. [13] and Simelane et al. [14], we found a positive association between improved toilet facility and HAZ of children. Unimproved sanitation was one of the key risk factors for poor nutritional outcome among under-five children in a study that analysed Demographic and Health Surveys from 137 developing countries [43]. Our finding is consistent with the study from Bangladesh that reported significant negative association between unimproved toilet facility and HAZ at lower quantile [12]. This implies that public health interventions aimed at improving household sanitation could benefit children at the lower quantiles of HAZ distribution more. The findings above demonstrate benefit of undertaking the quantile regression since such association could have been masked in standard mean-based regression.

Children belonging to Janajati and Newar were taller than those belonging to Brahmin, Chhetri and Madhesi. This result might be linked to the difference in infant and young child feeding practices between ethnic groups. Further research needs to be conducted to find out the concrete reasons behind this finding. We found that children born to mothers with age above 20 years are taller than those born to mothers with age below 20 years, one of the reasons to advocate against teenage pregnancy. Studies conducted in Bangladesh and Egypt have also found that the children born to mothers aged greater than 20 years tend to have better anthropometric measurement [3, 21]. However, contrary to these studies, our finding was statistically significant only at thirtieth and fiftieth percentiles. As discussed above, this relationship could also have been masked if only OLS regression was used.

Our finding is consistent with the findings from other studies that have found negative correlation between height of children and number of under-five children in household [14, 19]. Potential reasons behind this finding could be increased competition for food among siblings and household members and limited resources to address the household food demand [19, 41].

Children of Karnali, Sudurpaschim and Madhesh were most disadvantaged in terms of HAZ compared to Province 1. Geographical inequality in terms of public health and social protection indicators was evident in previous studies as well, especially in Karnali and Madhesh [28, 44–47]. In addition, Karnali and Madhesh were reported to have the highest rate of multidimensional poverty (50%), followed by Sudurpaschim (30%) [48]. Difficult geographical terrain, poor transportation facility and infrastructure, food insecurity, low overall socioeconomic development, lack of access to healthcare services, low levels of awareness are a few of the reasons behind the poor performance of these provinces [6, 45, 46, 49]. A community-based study conducted in an urban municipality of the mountainous Bajhang district of Sudurpaschim province has reported 54% of the households with food insecurity [50].

Similar to the study by Khan and Gulshan in Bangladesh [12], this study found HAZ of children increased monotonically in moving from the poorest wealth quintile to the richest wealth quintile at all quantiles except 30^th^. The inequality was more prominent at lower quantiles of HAZ distribution. Earlier studies from Nepal and other countries have consistently reported wealth-related inequality in health and nutritional outcomes in under-five children [3, 12, 17–19, 28, 38, 51, 52]. The possible reason behind this finding could be that the wealth status of a household has a direct link with affordability of required nutritious food, healthy living condition, and access to healthcare services [3, 12, 19, 21].

Recommendation for policymakers: Based on the findings from this study, a few recommendations could be made. First, different socioeconomic and demographic factors may have different effects on the nutritional status of children based on their position in the height-for-age distribution. So, targeted interventions are possible if the analysis is done taking the entire conditional HAZ distribution, and not just conditional mean. Second, interventions related to sanitation should be targeted to households with children at lower tails of HAZ distribution (children with low height-for-age). Third, children from Karnali, Madhesh and Sudurpaschim those at the lower tails of HAZ distribution could benefit more from nutritional interventions. Female Community Health Volunteers and community health workers should be mobilized to identify such children and deliver nutrition specific education [53]. Fourth, children at the lower tails of HAZ distribution and those belonging to poor households should be specifically targeted for nutrition intervention. Fifth, complementary feeding should be highly emphasized since children (no matter their position in the HAZ distribution) were at risk of undernutrition as they grow older. Sixth, the age of mother at birth (above 20 years) and education had a positive effect on nutritional outcome of children. Delay in marriage may provide women a chance to continue their education and prevent them from teenage pregnancy.

### Strength of this study

This study used the most recent nationally representative household survey from Nepal. Since MICS employs standard study design and tools to collect the data, the results are reliable. Given that previous studies from Nepal had predominantly used either standard linear or logistic regression, this study adds to the current body of literature by using more flexible econometric method that allowed a deeper understanding of strength of association and statistical significance between independent and dependant variables along the conditional quantiles of nutritional distribution. This approach enabled us to identify critical risk factors of under-five child nutrition that could not have been evident from OLS regression alone.

### Limitation of this study

We could not include potentially important variables related to maternal health characteristics (such as antenatal care visits, place of delivery) since such data was only available for mothers with the most recent live birth within two years preceding the survey. Similarly, we could not include height of mother due to unavailability of such data in the Nepal MICS 2019. Due to cross-sectional nature of the data, establishing a causal relationship was not possible. Notwithstanding, this study has elicited empirical evidence on socioeconomic and demographic correlates of nutritional status of children using flexible econometric methods. So, the findings presented in this study may have policy relevance to countries with similar socioeconomic contexts to Nepal.

## Conclusion

Both the OLS and quantile regression showed that age of children, sex of children, education status of mother, geographical province, and household wealth status were significant predictors of linear growth of children. Few variables emerged as significant predictors while performing quantile regression at different points: maternal age, ethnicity, improved water and sanitation facility, number of children in household, and number of household members. Use of quantile regression approach showed that the effect of different socioeconomic and demographic factors differed along the conditional distribution of HAZ. Our findings recommend that nutrition policy and intervention should consider the heterogeneous effect of different factors on HAZ so that targeted intervention could be implemented to maximize the nutritional benefits to children. A deeper understanding of the effect of different factors, for example, at the lower tail of the distribution, could be beneficial for policymakers in designing policies and interventions targeted towards the most disadvantaged children. Focused actions are imperative so that the targets of SDGs (ending all forms of malnutrition, UHC) could be achieved by 2030.

## Supplementary Information

**Additional file 1:**  **Supplementary Table 1. **Variance Inflation factors (VIFs) of variablesincluded in the multivariable regression model

## Data Availability

Publicly available data were used that are accessible from the MICS website (https://mics.unicef.org/surveys) upon request.

## Abbreviations

CI: Confidence interval
EA: Enumeration area
GoN: Government of Nepal
HAZ: Height-for-age z scores
MICS: Multiple indicator cluster survey
OLS: Ordinary least square
SD: Standard deviation
SDG: Sustainable development goal
UHC: Universal health coverage
UNICEF: United Nations Children's Fund
VIF: Variance inflation factor
WHO: World Health Organization
