# Supplementary material for: Socioeconomic and demographic correlates of child nutritional status in Nepal: an investigation of heterogeneous effects using quantile regression
Source: Global Health. 2022 Apr 20;18:42. doi: 10.1186/s12992-022-00834-4 (PMC9020427; doi:10.1186/s12992-022-00834-4)
Supplement: Supplementary file 1 — Additional file 1: Supplementary Table 1. Variance Inflation factors (VIFs) of variablesincluded in the multivariable regression model [file 12992_2022_834_MOESM1_ESM.docx]

**Socioeconomic and demographic correlates of child nutritional status in Nepal: an investigation of heterogeneous effects using quantile regression**

**Supplementary Information**

**Supplementary table 1** Variance Inflation factors (VIFs) of variables included in the multivariable regression model

| Variables | VIF |
| --- | --- |
| Child’s age (months) | 1.02 |
| Child’s sex (Male) | 1.01 |
| Birth order |  |
| Second and third | 1.41 |
| Fourth and above | 1.72 |
| Mother’s age at birth |  |
| 20-34 years | 1.54 |
| More than 34 years | 1.59 |
| Education status of mother |  |
| Primary education (grade1-5) | 1.52 |
| Secondary education (grade 6-10) | 2.17 |
| Higher secondary and above (grade 11 and above) | 2.35 |
| Number of under-five children in household |  |
| Two | 1.23 |
| Three | 1.39 |
| Four or more | 1.20 |
| Number of household members |  |
| Six to eight members | 1.27 |
| Nine or more members | 1.63 |
| Source of drinking water (unimproved) | 1.22 |
| Type of toilet facility (unimproved) | 1.12 |
| Ethnicity |  |
| Janajati and Newar | 1.53 |
| Dalit and Muslim | 1.25 |
| Others (eg. Marwadi, Bangali) | 1.23 |
| Residence (urban) | 1.23 |
| Province |  |
| Madhesh province | 2.39 |
| Bagmati province | 2.15 |
| Gandaki province | 1.39 |
| Lumbini province | 1.84 |
| Karnali province | 1.59 |
| Sudurpaschim province | 1.63 |
| Wealth index quintile |  |
| Poor | 1.90 |
| Middle | 1.98 |
| Richer | 2.20 |
| Richest | 2.68 |
| Mean | **1.61** |
